# The Periodontium as a Potential Cause of Orofacial Pain: A Comprehensive Review

**DOI:** 10.2174/1874210601812010520

**Published:** 2018-07-31

**Authors:** Jaume Miranda-Rius, Lluís Brunet-Llobet, Eduard Lahor-Soler

**Affiliations:** 1 Department of Odontostomatology, Faculty of Medicine and Health Sciences, Universitat de Barcelona, Barcelona, Spain; 2 Division of Orthodontics and Pediatric Dentistry, Hospital Sant Joan de Déu, Universitat de Barcelona, Barcelona, Spain; 3 Hospital Dentistry & Periodontal Medicine Research Group, Institut de Recerca Sant Joan de Déu (IRSJD), Fundació Sant Joan de Déu, Barcelona, Spain

**Keywords:** Orofacial pain, Gingival pain, Periodontal discomfort, Mucogingival herpetic lesions, Periodontal injury, Periodontal ligament strains

## Abstract

**Introduction:**

Orofacial pain of periodontal origin has a wide range of causes, and its high prevalence and negative effect on patients' quality of life make intervention mandatory. This review provides a periodontological overview of the field of orofacial pain, focusing on the entities which involve the periodontal tissues and may be the cause of this pain or discomfort.

**Methods:**

The study comprised a literature search of these pathologies conducted in the MEDLINE/PubMed Database. Acute infectious entities such as gingival and periodontal abscesses are emergencies that require a rapid response. Periodontitis associated with endodontic processes, necrotizing periodontal disorders, desquamative gingivitis, gingival recession, and mucogingival herpetic lesions, cause mild to severe pain due to tissue destruction and loss. Other lesions that lead to periodontal discomfort include gingival enlargement and periodontal ligament strains associated with occlusal trauma, parafunctional habit and the impaction of food or foreign bodies.

**Conclusion:**

A range of therapeutic, pharmacological and surgical alternatives are available for the management of these injuries. However, the wide variety of causes of orofacial pain or periodontal discomfort may confuse the clinician during diagnosis and may lead to the wrong choice of treatment.

## 1 INTRODUCTION

Orofacial pain is defined as pain associated with the hard and soft tissues of the head, face, mouth and neck. It may originate in dental, periodontal, vascular, glandular and muscular structures, and in bones, sinuses, and joints. This great variety of sources, each with its own complex innervation, explains the wide range of possible diagnoses in patients reporting orofacial pain [1, 2].

In dentistry, periodontal pain is relatively localized and therefore extends less than pain originating in the tooth pulp. The periodontal ligament is innervated by Aδ and C fibers which transmit sensations of pain and pressure. These fibers are involved in the exteroceptive function, although a distinction should be made between: **1-** nociceptors for pain, which perceive painful stimuli and produce reflex responses through the automatic opening of the mouth; **2-** mechanoreceptors that respond to the physical changes caused by touch and pressure; **3-** mechanoreceptors for proprioception that control the occlusal contacts and the masticatory function [3-5].

In periodontal structures, pain may be manifested in two different ways: pain of periapical origin, which is a deep somatic pain; or pain in the gingival tissue, which is a superficial somatic pain [3-5].

Chronic periodontal diseases such as gingivitis and periodontitis are usually painless, but they may cause mild, episodic or persistent dull pain due to inflammation or low-grade infection. In contrast, sharp periodontal pain is often associated with high-grade infection and inflammation [3, 4]. Some authors suggest that there may be mechanisms that act either independently or synergistically to produce a peripheral antinociceptive effect associated with these periodontal diseases [5].

The wide range of pathological conditions that can present with orofacial pain of periodontal origin may create confusion during diagnosis. In this paper, we present a comprehensive review of the pathologies that affect the periodontal support tissues and cause orofacial pain or discomfort.

## 2 METHODS

A literature search of the MEDLINE/PubMed database was carried out to identify original papers and reviews describing (**a**) orofacial pain, (**b**) orofacial pain associated with periodontal diseases, and (**c**) acute periodontal diseases. The search covered the following terms: periodontal (MeSH), gingival (MeSH), orofacial (MeSH), pain (MeSH), discomfort (MeSH) and “AND” - combined with terms “acute”, “disease,” “odontogenic myxoma,” “pyogenic granuloma,” “herpes virus”, “stomatitis”, “recession”, “mucogingival defect,” “endoperio lesion”, “trauma”, “enlargement”, and “abscess” (MeSH). The selected articles were reviewed by all the authors with a customized evaluation form for assessing orofacial pain of periodontal origin. This process was performed by consensus in three stages: **1)** assessment of the title alone; **2)** assessment of abstracts and removal of irrelevant citations; **3)** assessment of the full-text article.

## 3 PERIODONTAL PATHOLOGIES

Chronic gingivitis and periodontitis are the two most common entities in periodontology. However, a number of less frequent disorders also involve the periodontium and can cause orofacial pain or periodontal discomfort.

### 3.1 Gingival and Periodontal Abscesses

Gingival abscesses, which appear in the gingival sulcus, are comparatively rare. Periodontal abscesses usually occur in areas with periodontal pockets, in which deep spaces are generated around the teeth. They cause a dull, gnawing, localized pain but are not painful to percussion. The discomfort ranges from low intensity aches to severe acute pain. Periodontal abscesses may be tender to lateral periodontal pressure and the pain in the tooth adjacent to the injury usually worsens with chewing. Gingival abscesses are usually located in the marginal interdental tissue.

Both gingival and periodontal abscesses present localized reddening of the gingivae, which may include the alveolar mucosa. Injuries are fluctuant, violaceous, cyanotic or erythematous and may be accompanied by a fistula [3].

It is important to carry out differential diagnosis of the case of pulpal pain. Table **1** summarizes the main clinical features of irreversible pulpitis and periodontal abscesses. Abscesses are caused by the localized proliferation of periodontal bacterial flora in an affected area, but can also be caused by traumatic impaction of food or a foreign body. The microflora of the abscess predominantly contains periodontal pathogens including *Porphyromonas gingivalis*, *Prevotella intermedia*, *Fusobacterium nucleatum*, *Peptostreptococcus micros* and *Bacteroides forsythus* [6].

Areas with periodontal disease (periodontitis) present destruction of the connective tissue and alveolar bone around the tooth. Periodontitis is caused by the activation of host inflammatory mediators in response to bacterial microorganisms. The gingival or periodontal abscess is a focus of purulent exudate with granulation tissue, surrounded by infiltrating leukocytes [3].

For the diagnosis of periodontal abscess, inspection and examination by periodontal probe (to identify edema/gingival erythema, periodontal pockets and bone defects) and a radiographic image confirming the decrease in bone height, are usually sufficient. However, when the clinical features are not accompanied by periodontal disease and there is a history of trauma or impaction, gingival abscess is usually diagnosed [3].

Pericoronitis is an infection that is associated with an erupting tooth, especially the third molar. The mucosa covering the tooth becomes inflamed, with a traumatism caused by the opposing teeth and infection caused by food remnants under the mucosal layer. The pain is intense, and is usually associated with submandibular adenitis, sore throat, trismus and fever [7].

The treatment of gingival/periodontal abscess aims to achieve drainage of the purulent collection, either through an incision in the fluctuant area or through the periodontal pocket. In cases of periodontal abscess, root debridement is usually required, along with antibiotic and analgesic treatment [3, 7].

### 3.2 Periodontitis Associated with Endodontic Lesions

The term “endoperio” describes the relationship between pulpal and periodontal diseases. Endoperiodontal or pulpoperiodontal lesions are inflammatory lesions that simultaneously compromise the dental pulp and structures of the periodontal insertion [8].

Pulpal and periodontal diseases present certain common clinical symptoms such as sensitivity to percussion and inflammation. The two diseases can mimic each other both clinically and radiographically, and so an accurate diagnosis of the etiological factors involved is necessary for deciding the correct course of treatment. Gingival recession usually facilitates biofilm accumulation and localized inflammation, which usually makes dental brushing difficult and painful. Eventually the progressive destruction, periodontal insertion and bone loss surrounding the affected tooth may even induce pathological alterations in its own pulp tissue. Microbial substances and products released by the inflammatory process in the periodontium can access the pulp through the apical foramen, the exposed lateral, secondary and cavointerradicular ducts and the dentinal tubules exposed by wear of the root cementum. In periodontally affected teeth, inflammatory lesions of varying intensity have also been observed, including transitional states of inflammation, partial and total chronic pulpitis, and pulp necrosis [9, 10].

If the inflammation is confined to the pulp, the patient will describe the pain as intense and incessant because the neural portion of the pulp only transmits pain. However, if the inflammation has reached the periodontal ligament, it will be easier for the patient to determine the source of the pain, since this structure contains proprioceptive sensory fibers [10].

### 3.3 Necrotizing Periodontal Diseases

**Fig. (1):**
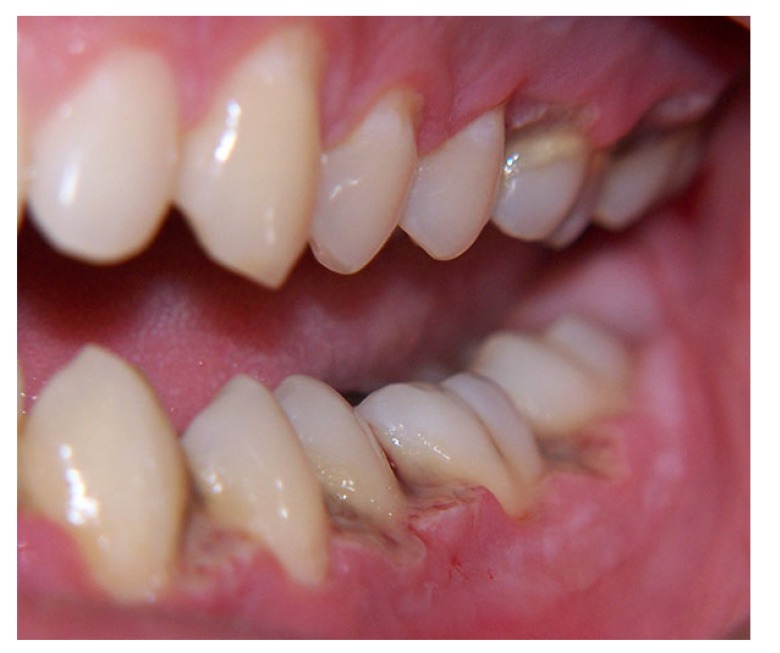
Necrotizing ulcerative gingivitis. Note the characteristic gingival papilla ulceration, crater defects and marginal pseudomembranes.

Necrotizing periodontal diseases are a group of infectious disorders comprising necrotizing ulcerative gingivitis, necrotizing ulcerative periodontitis and necrotizing stomatitis. However, these conditions may in fact present different stages of the same disease; their etiologies, clinical characteristics and treatments are similar but they differ in terms of severity [11]. The alterations present an acute inflammatory process and painful periodontal destruction [12]. The patient often complains of localized pain in the interdental papilla (Fig. **1**).

The main predisposing factors are stress, tobacco, alcohol, medication, drugs that cause a degree of xerostomia, poor diet, and lack of oral hygiene [13, 14]. However, necrotizing periodontal diseases have occasionally been reported with HIV infection [13, 14]. Treatment includes surgical debridement, chlorhexidine 0.20% (rinse) antibiotic therapy with amoxicillin + clavulanic acid and metronidazole.^13^ Necrotizing periodontal diseases can be classified as follows, according to their location [13-15]:

1. Necrotizing Ulcerative Gingivitis (NUG): Only the gingival tissue is affected. NUG is an acute, occasionally recurrent infection of complex etiology characterized by a sudden onset of interdental gingival pain, papilla necrosis, and bleeding. The change in the bacterial flora is the result of an altered host response, especially in adolescents and young adults (Fig. **1**).
2. Necrotizing ulcerative periodontitis: The necrosis progresses to the periodontal ligament and alveolar bone, thus undermining the insertion and support of the affected tooth. It may be a result of an episode of NUG that has evolved adversely, or it may occur at a site previously affected by periodontitis.
3. Necrotizing stomatitis: The necrosis progresses to deeper tissues beyond the mucogingival line, including the lip or the buccal mucosa, the tongue, and so on. Necrotizing stomatitis shares features with cancrum oris (or noma).

In all these lesions the onset of pain is rapid and its intensity depends on the extent of the injury. The episodes of pain increase during eating and tooth brushing, and are usually the reason for patient consultation [13-15].

The group of necrotizing periodontal diseases is characterized by the presence of highly severe inflammatory conditions associated with oral biofilm bacteria. These necrotic lesions can progress rapidly and cause severe tissue destruction. Therefore, a rapid response is vital, comprising periodontal treatment combined with effective oral hygiene measures and control of predisposing factors. However, relapses in NUG are frequent, usually due to a lack of control of the predisposing factors and the associated difficulty of achieving good control of the supragingival biofilm [11].

### 3.4 Gingival Recession, Mucogingival Defects and Periodontal Traumatic Injuries

Gingival recession reduces the width of keratinized connective tissue and may cause periodontal discomfort/slight pain, especially during dental brushing, due to localized inflammation and/or dentin hypersensitivity. It is associated with attachment loss due to the apical displacement of the gingival margin beyond the cementoenamel junction, resulting in exposure of the root surface [16]. The prevalence of gingival recession varies according to the population studied but it ranges between 30% and 100%. The main causes are plaque-induced inflammation and mechanical abrasion of soft and hard tissues. Other occasional causes are chemical and thermal injuries. Inside the mechanical etiology of gingival recession, traumatic brushing maintained over time also seems to be a contributing factor. This compulsive brushing is characterized by vigorous movements, causing painful microerosions of the mucosa and dentinal hypersensitivity [17-24].

**Fig. (2):**
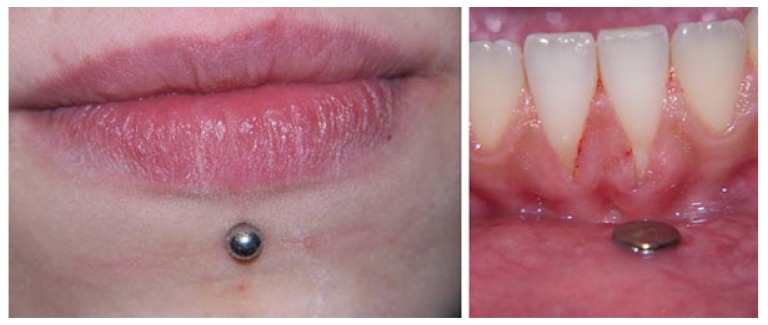
Lip piercing. Note the tooth gingival recession on the buccal area of the two central incisors. The intraoral piercing produced a chronic abrasive effect on the gingival margin.

Piercings in intra and perioral areas (in the lip, cheek and tongue, or frenum) may traumatize the surrounding tissue, resulting in localized chronic periodontitis. The control of associated factors is only possible with improved oral hygiene (Fig. **2**). The acute complications of piercings are associated with local infections, while late complications are mainly dental abrasion fractures and gingival recession of varying degrees of severity [25]. The management of gingival recession of mechanical origin centers on the elimination of the causal agent, suggesting to the patient new ways of toothbrushing, or recommending the removal of the piercing in the lip or the tongue. Depending on the Miller Class, various root coverage procedures may be performed using subepithelial connective tissue grafts [26].

### 3.5 Desquamative Gingivitis

Desquamative Gingivitis (DG) is a term that covers epithelial desquamation, erythema, erosion, and/or vesiculobullous lesions of the gingival. In DG, the gingivae are deep red in color, shiny, atrophic and eroded. There is a loss of the characteristic gingival pitting and it peels easily on minimum contact. Clinically there is a burning sensation or pain that is intensified by eating hot, acidic or spicy foods [27].

DG injuries mainly appear in the buccal aspect of the gingiva of the anterior teeth, especially with a diffuse pattern, but may occur anywhere in the gingiva. DG is frequently associated with muco-cutaneous alterations and systemic conditions with poor prognosis and high morbidity – among them oral lichen planus, oral lichenoid lesions, mucous membrane pemphigoid, pemphigus vulgaris and erythema multiforme [27].

Generally, the conditions associated with DG are most frequent between the fourth and sixth decades of life [28]. They are extremely rare in childhood and adolescence. DG is associated with female sex, especially during the menopause [29].

To treat this clinical condition, the use of a soft toothbrush and anti-plaque mouthwash is recommended, as well as the application of local anesthetic during maintenance periodontal treatment. Topical corticosteroids, in preparations such as triamcinolone acetonide 0.1% - 0.5% in orabase either with or without topical anesthetics such as lidocaine 1% and applied every 8/12h for 15 days, improve symptoms in the acute stages. Hydrocortisone in 2.5 mg tablets dissolved in the mouth every 6 h is another alternative [29].

### 3.6 Mucogingival Herpetic Lesions

Several viruses can cause lesions in the oral cavity. The ones most commonly associated with periodontal lesions are the family *Herpesviridae*: herpes simplex virus type 1, responsible for cold sores in the lip and the mouth; herpes simplex type 2, associated with genital herpes; the varicella zoster virus, responsible for chickenpox and herpes zoster) [30-33]. The primary infection by herpes simplex virus type 1 is usually asymptomatic, although it may sometimes manifest itself in the form of gingivostomatitis, causing widespread pain, dysphagia, fever, malaise and submandibular lymphadenopathies [34]. Recurrent infection by herpes type 1 is accompanied by local discomfort, is usually self-limiting, and only requires antivirals if it is severe. The differential diagnosis includes ulcers and aphthous stomatitis [35, 36].

The impact of the varicella zoster virus on the oral area is felt when the second (maxillary) or third (mandibular) branches of the trigeminal nerve are affected. Frequently, intraoral lesions which may extend to the periodontal tissues are accompanied by skin lesions in the area innervated by the sensory nerve affected. While some cases may begin with paresthesia of the mental nerve, the lesion usually starts with itching or burning along the nerve; after three or four days this is accompanied by the appearance of vesicles which then dry and form scabs within a period of 1-2 weeks. The intraoral vesicles appear after the ones in the skin, and are surrounded by an erythematous area which rapidly ulcers and is covered by a whitish pseudomembrane. The periodontal involvement in the more severe and rare cases may even be accompanied by rapid tooth exfoliation due to bone and/or periodontal ligament necrosis. The patient presents malaise and lymphadenopathies in the submandibular area [37].

The treatment of these injuries involves antiviral agents, analgesics, and antipyretics, with adequate nutritional support when feeding is compromised [34, 37].

## 4 OTHER ENTITIES RELATED TO PERIODONTAL DISCOMFORT

Gingival enlargement, defined as the abnormal overgrowth of gingival tissue, is another cause of periodontal discomfort. It is associated with multiple factors including inflammation, hormones, drug use, orthodontic forces, tumors, and genetic, systemic and idiopathic factors [38-43]. Although most gingival enlargements are generalized, this dimorphism may occasionally be localized as a result of a long-term proliferative and expansive process of the underlying structures: the jaw bone, periodontal ligament, and dental tissue [39, 44, 45].

Pyogenic Granuloma (PG) is a benign vascular lesion, which some authors describe simply as a non-infectious inflammatory hyperplasia characterized by fibrous and granulomatous tissue. Other authors define PG (also called epulis when located on the gingiva), as a tumor-like growth of the oral cavity that is considered non-neoplastic and is associated with a reparative or healing process [46]. Clinically, PG usually presents as a smooth or lobulated exophytic injury, either pedunculated or sessile [47, 48]. The surfaces of the injury may become ulcerated, and the inflammation may cause pain or discomfort [49]. For all these injuries, the treatment of choice is surgery in association with medical treatment with analgesics and anti-inflammatory agents [40, 42].

Another group of mechanical/physical injuries comprises those caused by food impaction, trauma due to occlusion and defective restorations, which may invade biologic width. For instance, the displacement of loosened or fractured obturations may cause them to be impacted in the periodontal soft tissues, thus provoking wounds and gingival abscesses [12]. Incorrect interproximal hygiene (*i.e.*, inadequate use of interdental brushes, toothpicks and dental floss) can also cause traumatic lesions such as ulcers and inflammation of the coronal area of the interdental papilla [50].

Finally, another situation that can lead to periodontal discomfort include periodontal ligament strains caused by occlusal trauma, associated with parafunctional habits [51]. High occlusal force is associated with localized worsening of the periodontal disease, increases in the space of the periodontal ligament, probe depth, bone loss mass and mobility, and discomfort especially in relation to function [52, 53].

## CONCLUSION

In medicine, all clinical findings must be submitted to differential diagnosis. This systematic process allows us to rule out entities that present with similar signs and symptoms, and thus obtain the definitive diagnosis.

An accurate medical history and clinical examination are essential in order to choose the appropriate therapeutic approach. Periodontal diseases associated with orofacial pain are common in clinical practice. They include infectious lesions, mucogingival disorders, gingival enlargement and periodontal trauma. In acute phases, these pathologies usually require emergency dental consultation.

In summary, a healthy periodontium with correct control of the supragingival biofilm does not just improve periodontal prognosis but can also reduce the number of consultations for orofacial pain or discomfort.

## Figures and Tables

**Table 1:** Differential diagnosis between pulpal and periodontal pain.

**Acute Irreversible Pulpitis**	**Periodontal Abscess**
Pain is not always localized	Pain is localized
Acute pain: piercing, throbbing and intermittent	Dull continuous pain. Uniform
Pain sensitive to changes in temperature	Pain not altered by changes in temperature
Painful to percussion	Not painful to percussion
Altered vitality tests	Normal vitality tests

Main features indicating final diagnosis of acute irreversible pulpitis and periodontal abscess.
